# Support Effects on the Activity of Ni Catalysts for the Propane Steam Reforming Reaction

**DOI:** 10.3390/nano11081948

**Published:** 2021-07-28

**Authors:** Aliki Kokka, Athanasia Petala, Paraskevi Panagiotopoulou

**Affiliations:** 1 School of Chemical and Environmental Engineering, Technical University of Crete, 73100 Chania, Greece; akokka@isc.tuc.gr; 2 Department of Chemical Engineering, University of Patras, 26504 Patras, Greece; natpetala@chemeng.upatras.gr

**Keywords:** propane steam reforming, H_2_ production, Ni, TiO_2_, CeO_2_, YSZ, ZrO_2_, Al_2_O_3_, drifts

## Abstract

The catalytic performance of supported Ni catalysts for the propane steam reforming reaction was investigated with respect to the nature of the support. It was found that Ni is much more active when supported on ZrO_2_ or YSZ compared to TiO_2_, whereas Al_2_O_3−_ and CeO_2_-supported catalysts exhibit intermediate performance. The turnover frequency (TOF) of C_3_H_8_ conversion increases by more than one order of magnitude in the order Ni/TiO_2_ < Ni/CeO_2_ < Ni/Al_2_O_3_ < Ni/YSZ < Ni/ZrO_2_, accompanied by a parallel increase of the selectivity toward the intermediate methane produced. In situ FTIR experiments indicate that CH_x_ species produced via the dissociative adsorption of propane are the key reaction intermediates, with their hydrogenation to CH_4_ and/or conversion to formates and, eventually, to CO, being favored over the most active Ni/ZrO_2_ catalyst. Long term stability test showed that Ni/ZrO_2_ exhibits excellent stability for more than 30 h on stream and thus, it can be considered as a suitable catalyst for the production of H_2_ via propane steam reforming.

## 1. Introduction

The high energy efficiency of fuel cells has drawn considerable attention toward the development of hydrogen production technologies. Hydrogen can be produced from fossil fuels either via hydrocarbon pyrolysis or hydrocarbon reforming processes including steam reforming, partial oxidation or autothermal reforming [1,2,3,4,5]. Biomass processes consisting of biological (bio-photolysis, dark fermentation, photo fermentation) and thermochemical (pyrolysis, gasification, combustion, liquefaction) methods as well as water splitting processes such as electrolysis, thermolysis or photolysis can be alternatively applied for the production of H_2_ from renewable energy sources [2,6]. However, the latter approaches are facing some major obstacles mostly related to high cost and low H_2_ yields. Currently, steam reforming of light hydrocarbons, including natural gas, ethane, propane, butane and liquified petroleum gas (LPG), are considered among the most promising and economical routes for hydrogen production [7]. Propane, which is the main component of LPG, has many advantages such as high energy density, compressibility to a transportable liquid at normal temperature and well-developed infrastructure which enable its use worldwide [8,9,10]. Moreover, propane can be stored and transferred as LPG through a wide distribution network or in high pressure cylinders in order to be supplied in remote places (e.g., agricultural, inaccessible or camping areas) or for domestic uses (e.g., households) [3,7].

Under propane steam reforming conditions, the water-gas shift reaction occurs simultaneously at low temperatures contributing to H_2_ and CO_2_ production, whereas CO/CO_2_ methanation may also run in parallel yielding CH_4_ and H_2_O. Methane can be also formed via hydrogenation of CH*_x_* species derived by the dissociative adsorption of propane on the catalyst surface or through propane decomposition accompanied by ethylene production. In certain cases, the C_2_H_4_, CH_4_ and CO thus produced are further decomposed leading to the formation of coke on the catalyst surface and consequently, to its progressive deactivation [11,12].

Supported noble metal (Rh, Ru, Pt, Pd) catalysts have been proposed to efficiently catalyze the production of H_2_ via propane steam reforming exhibiting high resistance to coke formation [3,11,13,14]. However, the high cost and low availability of noble metals are major drawbacks restricting their use in practical applications [15,16,17]. Noble metals can be replaced by nickel, which is less expensive and able to convert propane with high H_2_ yields. However, Ni is susceptible to coke formation and particles sintering considered to be responsible for catalyst deactivation [5,10,17]. The lifetime of Ni-based catalysts can be improved by optimization of the reaction conditions, catalyst promotion, improvement of the catalyst synthesis method as well as by the proper selection of the support [3,9,10,12,16,18,19].

Regarding the support material, it has been proposed that metal oxides characterized by high oxygen storage capacity, such as CeO_2_, YSZ, TiO_2_, ZrO_2_ or CeO_2_-ZrO_2_, are able to suppress carbon deposition through the participation of their lattice oxygen in carbon removal [3,16]. Moreover, Ni catalysts supported on metal oxides or promoted metal oxides, which favor steam adsorption and mobility of surface hydroxyls, have been found to facilitate coke gasification [17,20].

Metal-support interactions have been also reported to impose a dramatic effect on both carbon deposition and metal particles sintering under conditions of hydrocarbons reforming [5,21,22]. It was demonstrated that stronger interactions between Ni and MgAl_2_O_4_ support resulted in high Ni dispersion and inhibition of the formation of large Ni clusters [22], whereas weak interactions between Ni and SiO_2_ carrier were found to accelerate sintering and coke formation [21].

Therefore, the economic viability and practical applicability of H_2_ production via propane steam reforming may be facilitated by the development of efficient Ni catalysts supported on suitable metal oxides, which will be able to possess both high activity and resistance against carbon deposition and particles sintering to realize economically viable reforming processes. In the present study the effect of the nature of the support (TiO_2_, CeO_2_, Al_2_O_3_, YSZ, ZrO_2_) on the activity and selectivity of Ni-based catalysts for the propane steam reforming reaction was investigated. Mechanistic aspects related to the support influence on the reaction pathway were also studied and are discussed.

## 2. Materials and Methods

### 2.1. Catalyst Preparation and Characterization

The wet impregnation method was applied to prepare Ni (5 wt.%) catalysts supported on commercial metal oxide powders by using an aqueous solution of Ni (Ni(NO_3_)_2_∙6H_2_O) as the metal precursor salt. The commercial metal oxide carriers were used as received and were (a) activated aluminum oxide (Al_2_O_3_), catalyst support, 99% (metals basis) (Alfa Aesar, Kandel, Germany), (b) AEROXIDE^®^ TiO_2_ P25 (TiO_2_) (Evonik Industries AG, Essen, Germany), (c) cerium (IV) oxide (CeO_2_), nanopowder, 99.5% min (Alfa Aesar, Kandel, Germany), (d) yttria-stabilized zirconia (YSZ) (8Y-SZ, Tosoh, Amsterdam, The Netherlands) and (e) zirconium (IV) oxide (ZrO_2_) 99% (metal basis) (Alfa Aesar, Kandel, Germany). The resulting materials were dried at 110 °C for 24 h followed by reduction at 400 °C under H_2_ flow for 2 h. The selection of reducing Ni catalysts at 400 °C for 2 h was based on previous studies which indicated that under these reducing conditions nickel oxide species are able to be completely converted to metallic nickel [23,24,25].

The X-ray diffraction patterns of the catalysts were recorded using an X-ray powder diffractometer (A Brucker D8 Advance instrument, Bruker, Karlsruhe, Germany) using Cu *K_a_* radiation (*λ* = 0.15406 nm, 40 kV, 40 mA) and a scan rate of 0.025°/s over a range of 2*θ* between 20 and 80°. The diffraction pattern was identified by comparison with those supplied from the JCPDS data base, whereas the primary crystallite size of M_x_O_y_ (d_MxOy_) was estimated according to Scherrer’s equation:

(1) $$d_{M_{x}O_{y}}=\frac{0.9\cdot \lambda }{Β\cdot cos\theta }$$

where θ is the angle of diffraction corresponding to the peak broadening, B is the full-width at half maximum intensity (in radians) and λ = 0.15406 nm is the X-ray wavelength corresponding to Cu*K_a_* radiation.

The specific surface area (SSA) of the supported Ni catalysts were measured by N_2_ adsorption at 77 K (B.E.T. technique) using a Gemini III 2375 instrument (Micromeritics, Norcross, GA, USA). Carbon monoxide chemisorption measurements at 25 °C were applied for the determination of Ni dispersion and mean particle size using a modified Sorptomatic 1900 apparatus (Fisons Instruments, Glaskow, UK) and assuming a CO:Me stoichiometry of 1:1, an atomic surface area of 6.5 Å^2^ and spherical particles. CO chemisorption measurements were used instead of H_2_ chemisorption in order to avoid overestimation of Ni dispersion due to hydrogen spillover effects, which have been previously found to occur over supported Ni catalysts [26,27]. Nickel particle size was calculated according to the following equation:

(2) $$d_{Ni}=\frac{60000}{\rho_{Ni}\cdot S_{Ni}}\;[Å]$$

where d_N__i_ is the mean crystallite diameter, ρ_Ni_ (= 8.9 g∙cm^−3^) is the density of Ni and S_N__i_ [m^2^/g_Ni_] is the surface area per gram of Ni.

Transmission electron microscopy (TEM) images were obtained with a JEM-2100 system (JEOL, Akishima, Tokyo, Japan) operated at 200 kV (point resolution 0.23 nm) using an Erlangshen CCD Camera (Model 782 ES500W, Gatan Inc., Pleasanton, CA, USA). Samples were dispersed in water and spread onto a carbon-coated copper grid (200 mesh). Details related to the equipment and procedures used for catalyst characterization have been described in detail elsewhere [28].

### 2.2. Catalytic Performance Tests and Kinetic Measurements

The catalytic performance of the synthesized materials was studied in a tubular fixed-bed quartz reactor under atmospheric pressure using an apparatus which has been described in detail elsewhere [11]. The reaction conditions were as follows: temperature range 400–750 °C, H_2_O*/*C = 3.25, and gas hourly space velocity (GHSV) = 55,900 h^−1^. The reactor was loaded with 150 mg of catalyst (particle diameter: 0.15 < d_p_ < 0.25 mm) and placed in an electric furnace, where it was reduced in situ at 300 °C for 1 h under 50%H_2_/He flow (60 cm^3^ min^−1^) to ensure that the Ni exists in its metallic phase prior to catalytic performance tests. Catalyst reduction was followed by heating at 750 °C under He and subsequent switch of the flow to the feed stream consisted of 4.5%C_3_H_8_ + 0.15%Ar + 44%H_2_O (He balance). Argon was used as internal standard in order to account for the volume change. Water was fed through an HPLC pump (LD Class Pump, TELEDYNE SSI, PA, USA) into a vaporizer maintained at 180 °C and mixed with the gas stream coming from mass-flow controllers. A condenser immersed in an ice bath was placed at the exit of the reactor to condensate water prior to introduction of the gas stream to the analysis system. Reaction gases (He, 30%C_3_H_8_−1%Ar/He, H_2_) are supplied from high-pressure gas cylinders (Buse Gas, Bad Hönningen, Germany) and are of ultrahigh purity. Measurements of reactants’ and products’ concentrations were obtained by stepwise decreasing temperature up to 400 °C. The effluent from the reactor was analyzed using two gas chromatographs (Shimadzu, Kyoto, Japan) which were connected in parallel. The procedure used for gas phase analysis was described in our previous study [11]. The conversion of propane ($X_{C_{3}H_{8}}$) was calculated using the following expression:

(3) $$X_{C_{3}H_{8}}=\frac{{\left[Carbon\right]}_{total,out}^{}}{{\left[Carbon\right]}_{total,out}^{}+{\left[C_{3}H_{8}\right]}_{out}^{}}\times 100$$

where [Carbon]_total,out_ is the sum of the concentrations of all carbon containing products:

(4) $${\left[Carbon\right]}_{total,out}=\frac{\left[CO]+[CO_{2}]+[CH_{4}\right]}{3}+2\times \frac{\left[C_{2}H_{4}]+[C_{2}H_{6}\right]}{3}$$

Selectivity toward reaction products containing carbon was defined using Equation (5). The factor *n* corresponds to the number of carbon atoms in the corresponding molecule (e.g., for CO is 1, for C_2_H_4_ is 2 etc.):

(5) $$S_{Cn}=\frac{[C_{n}]\times n}{3\times {\left[Carbon\right]}_{total,out}^{}}\times 100$$

Selectivity toward hydrogen production was defined as the concentration of hydrogen produced divided with the concentration of all products containing hydrogen according to Equation (6). The factor m represents the number of hydrogen atoms in the corresponding molecule (e.g., for CH_4_ and C_2_H_4_ is 4).

(6) SH2(%)=[H2][H2]+m/2×[CHnm]×100

The intrinsic reaction rates for propane steam reforming reaction were measured for low propane conversions ($X_{C_{3}H_{8}}$< 10%) by varying W/F using the following expression:

(7) $$R_{C_{3}H_{8}}=\frac{{\left[C_{3}H_{8}\right]}_{in}^{}\cdot F_{in}-{\left[C_{3}H_{8}\right]}_{out}^{}\cdot F_{out}}{W}\times 100$$

where R_C3H8_ is the molar rate of C_3_H_8_ consumption (mol s^−1^ g_cat_^−1^), [C_3_H_8_]_in_, [C_3_H_8_]_out_, are the inlet and outlet concentrations (*v*/*v*) of C_3_H_8_, respectively, F_in_ and F_out_ are the total flow rates in the inlet and outlet of the reactor (mols^−1^), respectively, and W is the mass of catalyst (g_cat_).

Turnover frequencies (TOFs) of propane conversion were estimated following Equation (8) taking into account the measurements of both the reaction rates and nickel dispersions:

(8) $$TOF=\frac{R_{C_{8}H_{8}}\cdot AW_{Ni}}{D_{Ni}\cdot X_{Ni}}$$

where AW_Ni_ is the atomic weight of nickel (g_Ni_/mol_Ni_), X_Ni_ is the nickel loading (g_Ni_/g_cat_) and D_Ni_ is the dispersion of nickel.

### 2.3. In Situ FTIR Spectroscopy

In situ Fourier transform infrared (FTIR) experiments were carried out using an iS20 FTIR spectrometer (Nicolet, Thermo Fischer Scientific, Waltham, MA, USA) equipped with an MCT detector, a KBr beam splitter and a diffuse reflectance (DRIFT) sampling system (Specac, Orpington, UK) accompanied by an environmental chamber suitable for the study of diffusely reflecting solid samples in a controlled atmosphere. A flow system equipped with mass flow controllers, a steam saturator and a set of valves used for controlling the gas stream interacted with the catalyst surface, was directly connected to the gas inlet of the environmental chamber.

In a typical experiment, the catalyst powder was placed in the sampling system and heated at 500 °C in flowing helium for 10 min and then reduced under hydrogen flow at 300 °C for 30 min. The flow was then switched to He and the temperature was increased at 500 °C. After remaining 10 min at this temperature the sample was cooled at 100 °C. While cooling, the background spectra were recorded at the desired temperatures. Finally, the flow was switched to the reaction mixture, which consisted of 0.5%C_3_H_8_ +5%H_2_O (in He). Steam was introduced to the system via an independent He line passing through a saturator containing water maintained at 60 °C. The resulting gas mixture was fed to the DRIFT cell through stainless steel tubing maintained at 60 °C by means of heating tapes. A spectrum was collected at 100 °C after 15 min-on-stream followed by a stepwise increase of temperature up to 500 °C. During heating, spectra were recorded at selected temperatures after an equilibration for 15 min. In all experiments, the total flow through the DRIFT cell was 30 cm^3^ min^−1^. Reaction gases (He, 2%C_3_H_8_/He, H_2_) are supplied from high-pressure gas cylinders (Buse Gas, Bad Hönningen, Germany) and are of ultrahigh purity.

## 3. Results

### 3.1. Catalyst Characterization

**Figure 1:**
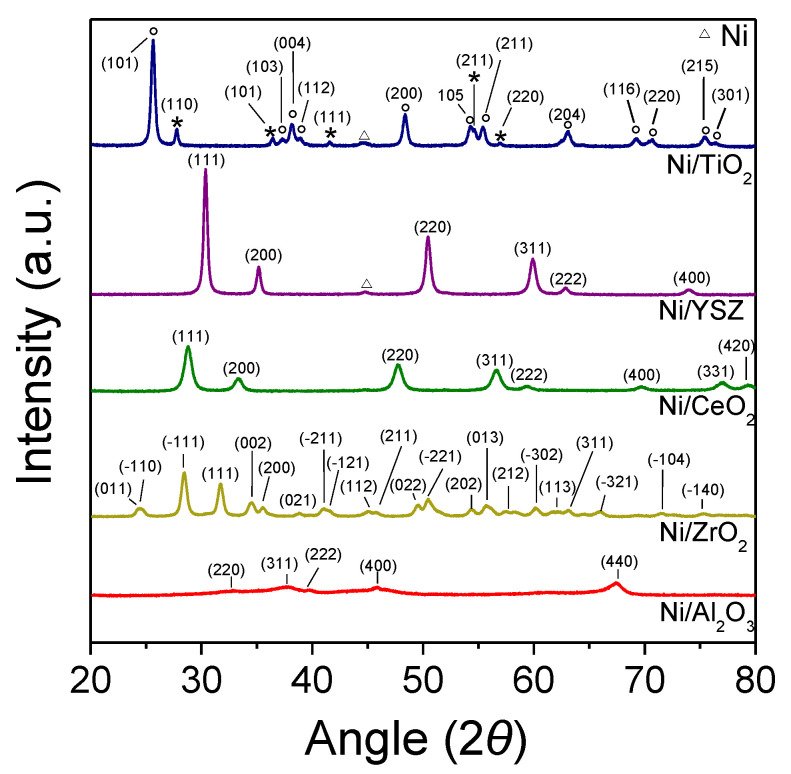
XRD patterns obtained from 5 wt.% Ni catalysts supported on the indicated commercial metal oxide carriers. The reflection planes of anatase (°) and rutile (*) TiO_2_ phases are indicated in the diffractogram of Ni/TiO_2_ catalyst.

The XRD patterns of Ni/M*_x_*O*_y_* catalysts are shown in Figure 1. The characteristic peaks located at the diffraction angles of 32.7°, 37.7°, 39.9°, 45.8° and 67.5° were appeared for Ni/Al_2_O_3_ which are attributed to (220), (311), (222), (400) and (440) facets of cubic Al_2_O_3_ (JCPDS Card No. 10-425), respectively. The XRD spectra recorded for Ni/CeO_2_ catalyst consisted of peaks located at 2θ = 28.79°, 33.26°, 47.62°, 56.34°, 59.1°, 69.42°, 76.66°, 79.11° attributed to (111), (200), (220), (311), (222), (400), (331) and (420) planes of the cubic CeO_2_ (JCPDS Card No. 2-1306), whereas in the case of Ni/ZrO_2_ catalyst numerous peaks were detected on the XRD pattern. In particular, the peaks detected at 24.2°, 24.5°, 28.5°, 31.7°, 34.5°, 35.5°, 38.9°, 41.0°, 41.5°, 45.1°, 45.8°, 49.5°, 50.5°, 54.4°, 55.7°, 57.5°, 60.2°, 62.1°, 63.2°, 66.0°, 71.6°, 75.3° correspond to (011), (−110), (−111), (111), (002), (200), (021), (−211), (−121), (112), (211), (022), (−221), (202), (013), (212), (−302), (113), (311), (−321), (−104), (−140) planes of monoclinic ZrO_2_ (JCPDS Card No. 13-307). When Ni was supported on YSZ, the XRD pattern was characterized by reflections at 30.4°, 35.1°, 50.4°, 59.9°, 62.9° and 74.0° attributed to (111), (200), (220), (311), (222) and (400) planes of YSZ (JCPDS Card No. 82-1246), respectively. 

Results obtained from Ni/TiO_2_ catalyst showed that the sample consisted of TiO_2_ in both its anatase and rutile form exhibiting peaks at 2θ = 25.6° (101), 37.2° (103), 38.2° (004), 38.9° (112), 48.4° (200), 54.3° (105), 55.4° (211), 63.1° (204), 69.3° (116), 70.7° (220), 75.4° (215) and 76.4° (301) for anatase (JCPDS Card No. 21-1272), and at 2θ = 27.6° (110), 36.3° (101), 41.6° (111), 44.3° (210), 54.6° (211), 56.9° (220) and 64.3° (310) for rutile (JCPDS Card No. 21-1276).

In the case of Ni/TiO_2_ and Ni/YSZ catalysts an additional weak peak located 44.5° was appeared corresponding to Ni (111) plane (JCPDS Card No. 04-0850). The absence of peaks corresponding to metallic Ni for the rest catalysts investigated is due to the low Ni loading and/or particle size. The primary crystallite size of the supports was estimated according to Scherrer’s formula at the diffraction angles corresponding to (440) plane for Al_2_O_3_, (−111) plane for ZrO_2_, (111) plane for CeO_2_, (111) plane for YSZ and (101) plane for TiO_2_, and it was found to be 6.0 nm for Ni/Al_2_O_3_, 10.5 nm for Ni/CeO_2_, 15.0 nm for Ni/ZrO_2_, 20.9 nm for Ni/YSZ and 21.8 nm for Ni/TiO_2_ (Table 1).

The SSAs of Ni catalysts supported on metal oxide (M*_x_*O*_y_*) carriers were estimated equal to 39 m^2^/g for Ni/ZrO_2_, 11 m^2^/g for Ni/YSZ, 66 m^2^/g for Ni/Al_2_O_3_, 39 m^2^/g for Ni/CeO_2_ and 41 m^2^/g for Ni/TiO_2_ (Table 1).

Results of Ni dispersion (D_Ni_) and mean particle size (d_Ni_) estimated from CO chemisorption meaurements are summarized in Table 1. Generally, low Ni dispersions were estimated for all the investigated catalysts, most possibly due to the high Ni content (5 wt.%) in agreement with previous studies [4,6]. Higher Ni dispersion of 11.9% and smaller particle size of 8.5 nm was found for Ni/CeO_2_ catalyst, whereas Ni/TiO_2_ exhibited the lowest value of Ni dispersion of 2.8% and the largest particle size of 36.1 nm.

**Figure 2:**
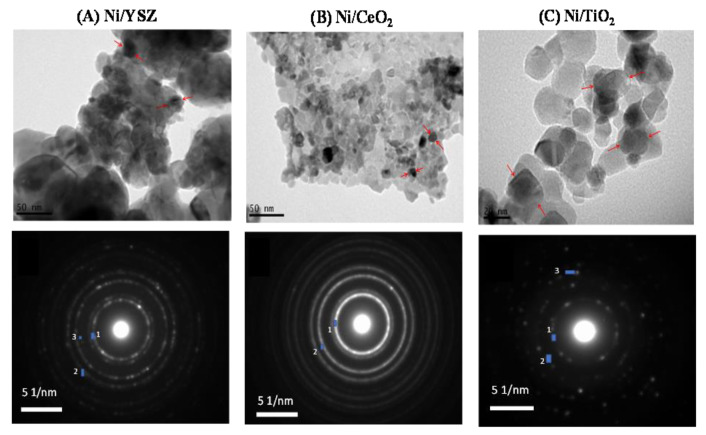
TEM images and Selected Area Electron Diffraction (SAED) patterns obtained for (**A**) 5%Ni/YSZ, (**B**) 5%Ni/CeO_2_ and (**C**) 5%Ni/TiO_2_ catalysts. Ni particles are indicated with red arrows.

Figure 2 shows representative TEM images and selected area electron diffraction (SAED) patterns obtained from Ni/YSZ, Ni/CeO_2_ and Ni/TiO_2_ catalysts. In all cases Ni particles appear as fairly homogeneously distributed spherical particles with average sizes of 20 nm for Ni/YSZ, 10 nm for Ni/CeO_2_ and 30 nm for Ni/TiO_2_, in agreement with those estimated according to CO chemisorption measurements (Table 1).

It should be noted that, based on the results of Table 1, the mean particle size of Ni is similar to the average size of the corresponding metal oxide used as support for all the investigated catalysts. This may hinder distinguishing between Ni particles and the M_x_O_y_ carrier in TEM images. Thus, SAED analysis was performed to calculate the d-spacing in an attempt to further discerned Ni particles from those of metal oxide support (Figure 2, Table 2). Results indicated that, in all cases, Ni particles are present in TEM images as evidenced by the appearance of the (111) plane of Ni (d_spacing_ = 0.21 nm, JCPDS No 1-1258). The appearance of the (101) (d_spacing_ = 0.35 nm, JCPDS No 1-562) and (103) (d_spacing_ = 0.24 nm, JCPDS No 1-562) planes of TiO_2_, the (111) (d_spacing_ = 0.30 nm, JCPDS No 30-1468) and (200) (d_spacing_ = 0.26 nm, JCPDS No 30-1468) planes of YSZ, as well as the (111) (d_spacing_ = 0.31 nm, JCPDS No 1-800) and (200) (d_spacing_ = 0.27 nm, JCPDS No 1-800) planes of CeO_2_ also confirmed the presence of the metal oxides.

### 3.2. Influence of the Nature of the Support on Catalytic Activity

**Figure 3:**
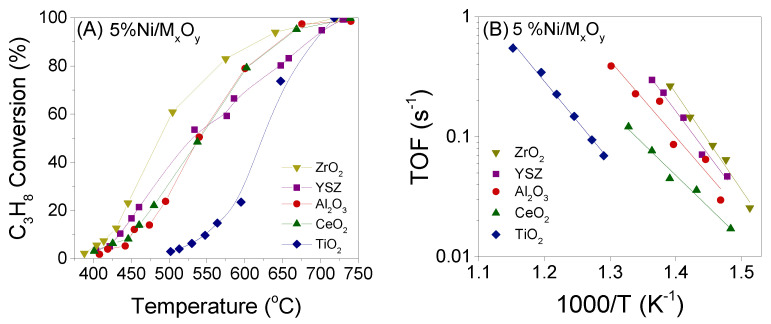
(**A**) Conversions of C_3_H_8_ as a function of reaction temperature and (**B**) Arrhenius plots of turnover frequencies of C_3_H_8_ conversion obtained over Ni catalysts (5.0 wt.%) supported on the indicated commercial oxide carriers. Experimental conditions: Mass of catalyst: 150 mg; particle diameter: 0.15 < d_p_ < 0.25 mm; Feed composition: 4.5% C_3_H_8_, 0.15% Ar, 44% H_2_O (balance He); Total flow rate: 250 cm^3^ min^−1^.

The influence of the nature of the support on catalytic performance for the propane steam reforming reaction has been investigated using Ni catalysts (5 wt.%) supported on five different commercial metal oxide powders (ZrO_2_, YSZ, TiO_2_, Al_2_O_3_, CeO_2_). The results obtained are shown in Figure 3A, where propane conversion is plotted as a function of reaction temperature. It is observed that, among the investigated catalysts, Ni/ZrO_2_ is the most active one, exhibiting measurable C_3_H_8_ conversions at temperatures higher than 400 °C and achieving complete conversion at 750 °C. Although Ni/YSZ is activated at similar temperatures as Ni/ZrO_2_, the conversion curve of propane is shifted toward higher temperatures. This is also the case for Ni/Al_2_O_3_ and Ni/CeO_2_ catalysts, which present similar performance. The latter catalysts are less active than Ni/YSZ below 550 °C, but are able to reach higher *X*_C3H8_ at higher temperatures. The titania-supported catalyst becomes active above 500 °C, with the propane conversion curve being shifted at remarkably higher temperatures. In all examined cases, the carbon balance was satisfactory, with a deviation of <1%.

Results of specific reaction rate measurements are presented in the Arrhenius diagram of Figure 3B, where it is observed that the TOF of propane conversion increases in the order Ni/TiO_2_ < Ni/CeO_2_ < Ni/Al_2_O_3_ < Ni/YSZ < Ni/ZrO_2_, with its value at 450 °C being more than one order of magnitude higher when Ni is dispersed on ZrO_2_ compared to TiO_2_, and approximately 2.5 times higher than that of Ni/Al_2_O_3_. It should be mentioned that, as discussed above, the mean particle size of Ni varies significantly for the investigated catalysts from 8.5 nm for Ni/CeO_2_ to 36.1 nm for Ni/TiO_2_. If the Ni particle size were similar for this set of catalysts then the order of catalytic activity could be somewhat different. Interestingly, no trend was observed between the specific reaction rate and Ni particle size or M_x_O_y_ crystallite size or M_x_O_y_ surface area. This indicates that either these parameters do not affect catalytic activity or most possibly each of them contributes in a different manner to the reaction rate, resulting in the observed catalyst ranking. It should be noted that all catalysts have been reduced at 400 °C prior to physicochemical characterization measurements. Although the values of SSA or d_MxOy_ or d_Ni_ may were different if catalyst pre-reduction was carried out at 750 °C, which is the onset reaction temperature for catalytic performance experiments, the trend of catalytic properties with respect to the nature of the support is not expected to vary due to the catalyst pretreatment at different temperatures, at least to such an extent that would affect the catalyst ranking for the propane steam reforming reaction.

The apparent activation energies (E_a_) of the propane steam reforming reaction were calculated from the slopes of the fitted lines of Figure 3B. The results showed that the nature of the metal oxide carrier significantly affects E_a_, which takes values between 102 kJ/mol for Ni/CeO_2_ and 154 kJ/mol for Ni/ZrO_2_ without presenting any trend with respect to catalytic activity (Table 1). This can be explained taking into account that, as it will be discussed below, several reactions run in parallel under the present experimental conditions each one of which is influenced by the nature of the support in a different manner resulting in the observed random variation of E_a_ with catalytic activity. The results are in agreement with our previous study where it was found that the apparent activation energy for the reaction of steam reforming of propane over Rh catalysts supported on a variety of metal oxides does not present any trend with the activity order [11].

**Figure 4:**
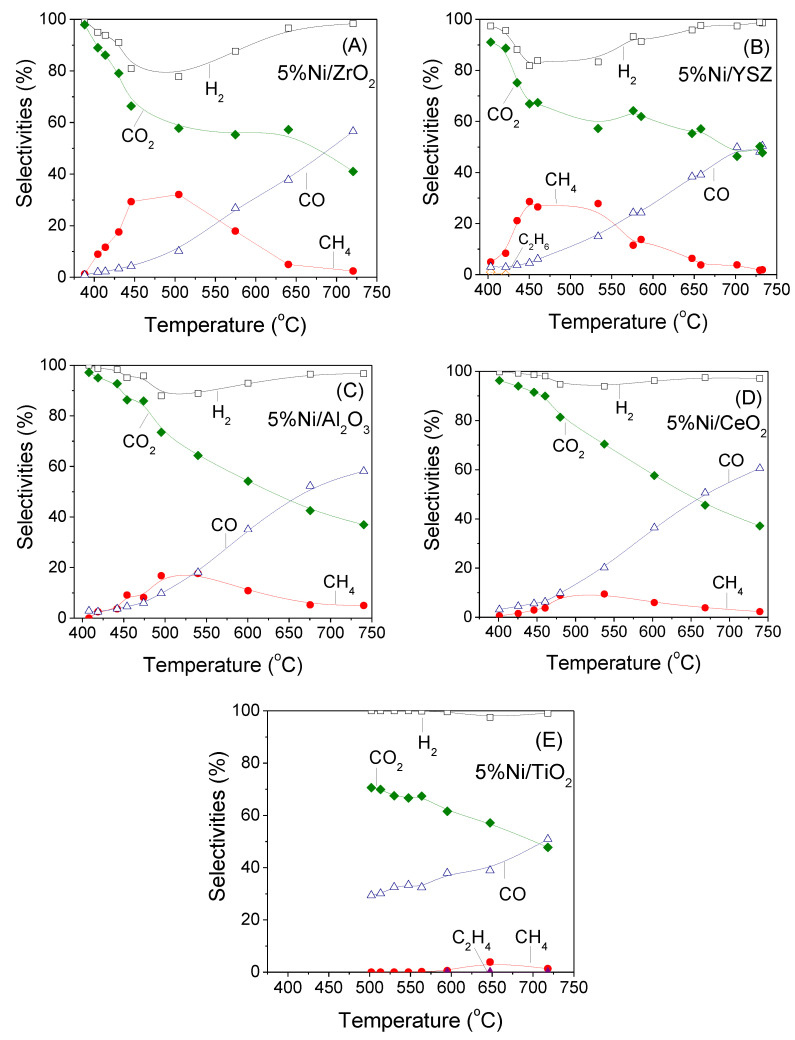
Selectivities toward reaction products as a function of reaction temperature obtained over Ni catalysts (5.0 wt.%) supported on (**A**) ZrO_2_, (**B**) YSZ, (**C**) Al_2_O_3_, (**D**) CeO_2_ and (**E**) TiO_2_. Experimental conditions: same as in Figure 3.

Figure 4 shows the selectivities toward reaction products as a function of temperature over the supported Ni catalysts investigated. In all cases the main products detected were H_2_, CO_2_, CO and CH_4_ with their selectivities being significantly varied with temperature. In particular, for Ni/ZrO_2_ catalyst (Figure 4A), both hydrogen (*S*_H2_) and CO_2_ (*S*_CO2_) selectivities decrease from 99 to 78% and from 98 to 58%, respectively, with increasing temperature in the range of 390–505 °C followed by an increase of methane selectivity (*S*_CH4_) up to 32.5%, indicating the occurrence of CO_2_ methanation reactions. Carbon dioxide consumption continues with further increase of temperature above 505 °C contrary to *S*_H2_ which progressively increases reaching 99% at 720 °C. Consumption of CO_2_ is followed by production of CO providing evidence that the reverse WGS (RWGS) reaction is enhanced at high temperatures. Moreover, *S*_CH4_ decreases above 505 °C and becomes practically zero at 720 °C, implying that the reaction of methane steam reforming occurs contributing to the observed increase of both *S*_H2_ and *S*_CO_. It should be noted that selectivity toward reaction products containing carbon was defined as the concentration of each product containing carbon at reactor effluent over the concentration of all products containing carbon (5), whereas S_H2_ was defined as the concentration of hydrogen produced divided by the concentration of all products containing hydrogen (6). Therefore, the values of *S*c_n_ and *S*_H2_ cannot be correlated based on the stoichiometry of the reactions taking place under propane steam reforming conditions.

**Figure 5:**
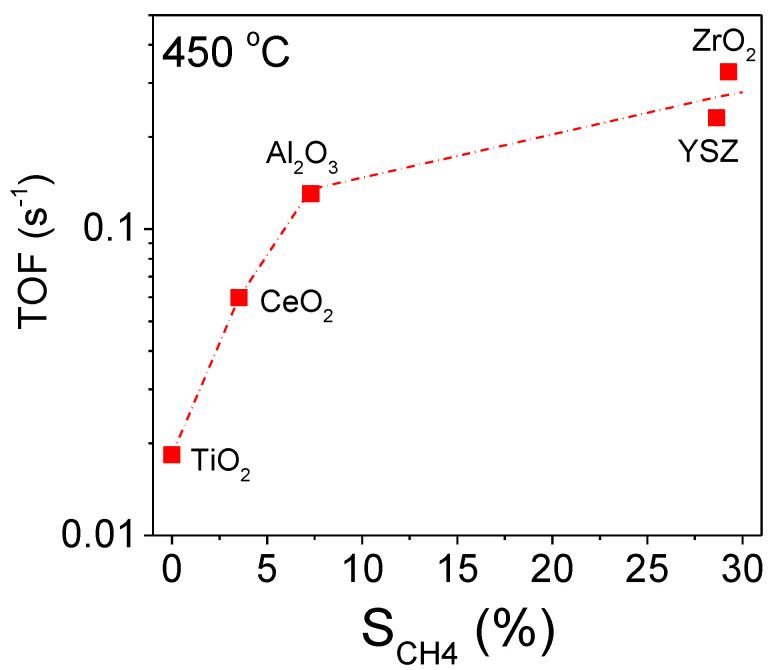
Turnover frequencies of C_3_H_8_ conversion as a function of selectivity toward CH_4_ obtained at 450 °Cover supported Ni catalysts.

Qualitatively similar results were obtained for the rest of the investigated Ni catalysts, with the main differences being related to the values of the selectivities toward the reaction products, which reflect the extent of each reaction taking place with respect to the nature of the support. In particular, the observed decrease of *S*_H2_ and *S*_CO2_ at low temperatures and the simultaneous increase of *S*_CH4_ are higher for the most active Ni/ZrO_2_ (Figure 4A) and Ni/YSZ (Figure 4B) catalysts, followed by Ni/Al_2_O_3_ (Figure 4C) and Ni/CeO_2_ (Figure 4D), whereas it is eliminated for Ni/TiO_2_ (Figure 4E). As a result methane production increases in the order of Ni/TiO_2_ < Ni/CeO_2_ < Ni/Al_2_O_3_ < Ni/YSZ < Ni/ZrO_2_ which is consistent with the order of catalytic activity. This can be clearly seen in Figure 5 where the TOF at 450 °C is plotted as a function of methane selectivity obtained at the same temperature for all the investigated catalysts. It is observed that the specific reaction rate increases from 0.018 s^−1^ to 0.33 s^−1^ following the above catalyst ranking and accompanied by a parallel increase of *S*_CH4_ from 0 to 29%. The results indicate that there is a clear relationship between catalytic activity and methane production.

It should be noted that besides CO_2_ hydrogenation, CH_4_ can be also produced via CO hydrogenation. However, the contribution of the latter reaction does not seem to be significant for the results of the present study taking into account the low *S*_CO_ below 500 °C and its progressive increase with temperature. Moreover, methane formation may also take place via hydrogenation of CH*_x_* species formed following the dissociative adsorption of propane on Ni surface and the subsequent hydrogenation of the so-formed C_3_H_x_ species [29,30]. As it will be discussed below, the formation of CH_x_ species intermediates may be the key reaction since it has been proposed that they interact with the hydroxyl groups or lattice oxygen of the support producing CO or CO_2_ and H_2_ [3,29,30].

The mass of H_2_ produced per propane mass unit contained in the feed was calculated at 550 °C and it was found to be higher for Ni/ZrO_2_ (23.2 wt.%) followed by Ni/CeO_2_ (21.2 wt.%), Ni/Al_2_O_3_ (20.6 wt.%) and Ni/YSZ (19.7 wt.%), whereas Ni/TiO_2_ exhibits the lowest H_2_ production (5.2 wt.%).

The influence of the nature of the support on the activity of Ni catalysts for propane steam reforming reaction was also investigated by Harshini et al. [16], who found that Ni/LaAlO_3_ was more active than Ni/Al_2_O_3_, while Ni/CeO_2_ exhibited intermediate performance. The optimum activity of the former catalyst was attributed to the small Ni nanoparticles dispersed on LaAlO_3_ surface. Although the effect of the support nature on propane steam reforming activity has not been widely studied over Ni catalysts, certain properties of metal oxide carriers may help explain the results of Figure 3. For example, the use of YSZ as support, which exhibited high activity in the results of the present study, has been found to suppress carbon deposition over Rh-Ni catalysts by providing lattice oxygen, which facilitates carbon removal and enhances the dissociation of C-C bond under reaction conditions [3]. The prevention of coke formation, occurring either via hydrocarbons decomposition or CO dissociation, by the lattice oxygen of the support has been also demonstrated over Ni/CeO_2_-Al_2_O_3_ [19]. Moreover, the addition of manganese oxide on Ni/Al_2_O_3_ was found to act as an oxygen donor that is transferred to Ni particles leading to rapid decomposition and oxidation of C_3_H_8_ and CH_4_ or C_2_H_4_ that may be produced under reaction conditions, resulting in further H_2_ production and improvement of the catalyst lifetime [9]. It has been also found that activation of steam followed by H_2_ formation may be favored over metal catalysts supported on “reducible” metal oxides through generation of oxygen defects, resulting in improved propane steam reforming activity and resistance to coke formation [3,13,31]. Based on previous studies, the reducibility of the supports used in the present study is expected to vary significantly. It is well known that Al_2_O_3_ is a hardly reducible metal oxide characterized by low oxygen storage capacity contrary to TiO_2_ and CeO_2,_ which are strongly reducible metal oxides, or ZrO_2_ and YSZ, which are characterized by intermediate oxygen mobility [32]. Based on the above, Ni/TiO_2_ should be also active for the title reaction, taking into account that titania support is characterized by high oxygen storage capacity [32,33]. However, the results of Figure 3 clearly show that: (a) this catalyst was the least active one and (b) the catalytic activity is increased in the order Ni/TiO_2_ < Ni/CeO_2_ < Ni/Al_2_O_3_ < Ni/YSZ < Ni/ZrO_2_, which cannot be correlated with the reducibility of the support. Therefore, it is evident that support reducibility is not among the key parameters affecting the catalytic activity of Ni according to the results of the present study. The low activity of Ni/TiO_2_ catalyst may be related to the fact that Ni/TiO_2_ has lower Ni dispersion and larger Ni particles, which was previously suggested to suppress both propane steam reforming [15,16], and the intermediate (CO_2_ or CH_x_) hydrogenation reactions, in excellent agreement with the results of our previous study [28]. However, large Ni particles may not be solely responsible for the low activity of Ni/TiO_2_ taking into account that no trend was observed between TOF and Ni particle size for the investigated catalysts.

It should be mentioned that a different activity order was reported in our previous study over supported Rh catalysts, where it was found that Rh/TiO_2_ was the most active catalyst with TOF being one order of magnitude higher compared to that measured for Rh/CeO_2_ [11]. This implies that the nature of the metallic phase may affect metal/support interactions leading to variations on propane steam reforming activity and/or possibly changes on the type of active sites on the catalyst surface.

### 3.3. Long Term Stability Test

**Figure 6:**
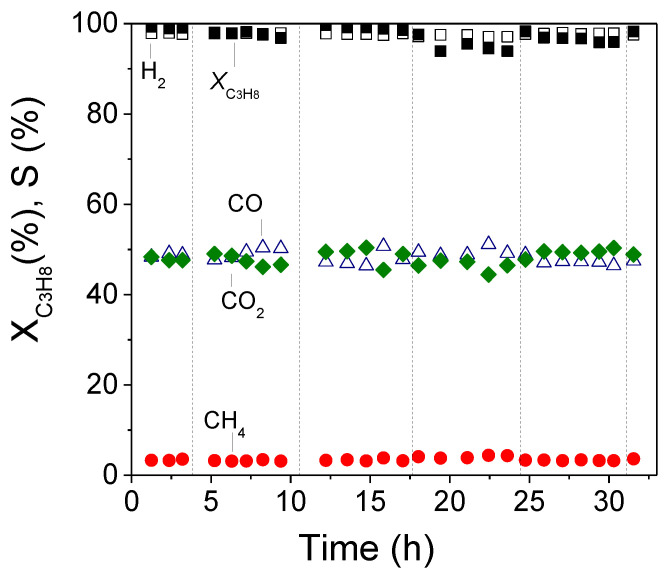
Long-term stability test of the 5%Ni/ZrO_2_ catalyst at 650 °C: Alterations of the conversion of C_3_H_8_ and selectivities toward reaction products with time-on-stream. Experimental conditions: Same as in Figure 3. Dashed vertical black lines indicate shutting down of the system overnight.

The long-term stability of Ni/ZrO_2_ catalyst, which exhibited the highest activity, was investigated at 650 °C using the same experimental conditions as those used in catalytic performance tests. In this experiment, the catalyst was reduced in situ at 300 °C under 50%H_2_/He flow followed by heating at 650 °C. The flow was then switched to the reaction mixture and determination of the conversion of propane and product selectivity started. The system was shut down overnight, while the catalyst was kept at room temperature under a He flow. The next day the catalyst is heated to 650 °C in the He flow, followed by switching of the flow to the reaction mixture and determination of *X*_C3H8_ and the product selectivity as a function of time. Results obtained are shown in Figure 6, where *X*_C3H8_ and *S*_H2_, *S*_CO2_, *S*_CO_ and *S*_CH4_ are plotted as functions of time-on-stream. As it can be seen the catalyst presents excellent stability for more than 30 h-on-stream. Propane conversion and hydrogen selectivity were varied in the range of 95–99% and 97–98%, respectively. The selectivity toward methane was low (3–4%) whereas *S*_CO_ and *S*_CO2_ exhibited similar values ranging between 46 and 50%. The carbon balance was found to be satisfactory during the stability test, with a deviation lower than 1–2%.

### 3.4. DRIFT Studies

**Figure 7:**
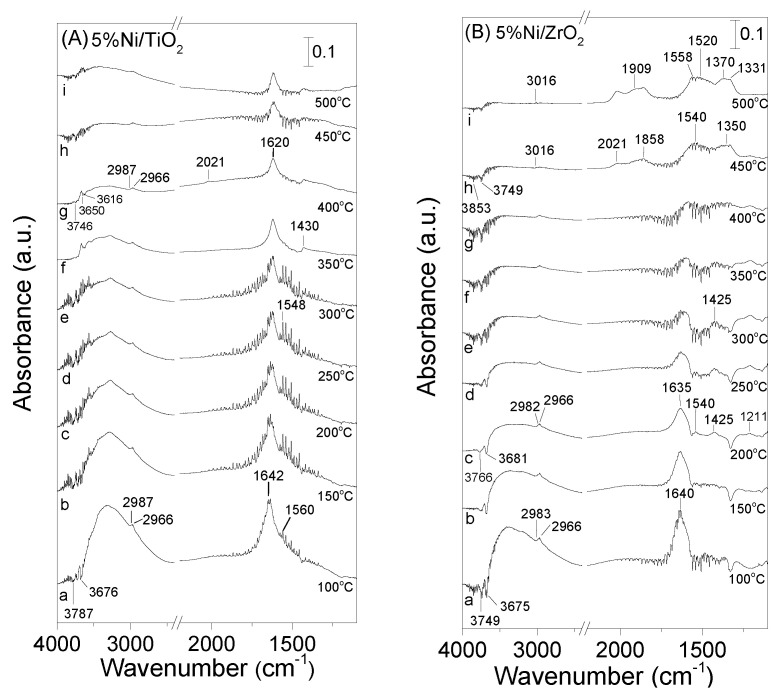
DRIFT spectra obtained over the (**A**) Ni/TiO_2_ and (**B**) Ni/ZrO_2_ catalysts following interaction with 0.5% C_3_H_8_ + 5% H_2_O (in He) at 100 °C for 15 min and subsequent stepwise heating at 500 °C.

**Figure 8:**
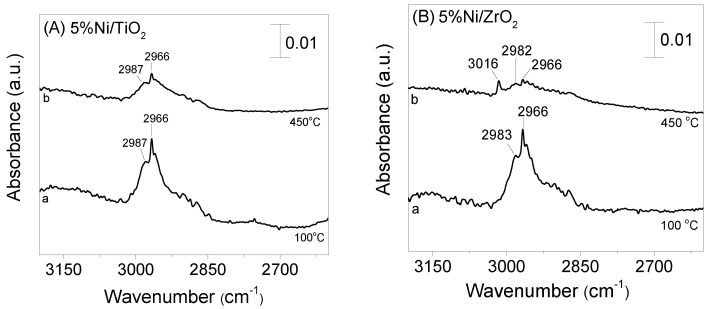
DRIFT spectra obtained in the region 3200–2400 cm^−1^ over the (**A**) Ni/TiO_2_ and (**B**) Ni/ZrO_2_ catalysts following interaction with 0.5% C_3_H_8_ + 5% H_2_O (in He) at 100 °C and 450 °C for 15 min.

The interaction of selected catalysts with the reaction mixture was also investigated employing in situ FTIR spectroscopy. Experiments were conducted in the temperature range of 100–500 °C using a feed composition of 0.5%C_3_H_8_ + 5%H_2_O (in He) and the results obtained are shown in Figure 7. It is observed that the spectrum recorded at 100 °C (Figure 7A, trace a) for the pre-reduced Ni/TiO_2_ catalyst is characterized by two negative bands at 3787 and 3676 cm^−1^ which can be attributed to losses of *ν* (OH) intensity of at least two different types of free hydroxyl groups, which are either originally present on TiO_2_ surface or created via H_2_O adsorption. Two weak peaks were also detected in the *ν* (C-H) region, located at 2987 and 2966 cm^−1^ (trace a) due to C-H stretching vibrations in methyl groups (CH_3,ad_) and to symmetric C-H vibrations in methylene groups (CH_2,ad_), respectively [20,31,34,35,36]. These peaks are more obvious in Figure 8A (trace a) where selected spectra in the narrow range of 3200–2400 cm^−1^ are presented. Moreover, a band at 1642 cm^−1^ followed by a shoulder at 1560 cm^−1^ can be discerned, which have been previously assigned to carbonate species associated with TiO_2_ support [37,38,39,40,41,42]. An increase of temperature results in progressive separation of the latter two bands which are both shifted toward lower wavenumbers. A new band at 1430 cm^−1^ can be also discerned in the spectra obtained at 350 °C (trace f) which is also due to carbonate species. This peak may be also present in the spectra obtained at lower temperatures but couldn’t be clearly observed due to the low signal-to-noise ratio in the region below 1700 cm^−1^. The intensities of bands assigned to carbonate species are progressively decreased above 200 °C. This decrease is accompanied by the detection of a weak peak at 2021 cm^−1^ [38,43,44,45,46], which is characteristic of linear-bonded CO on reduced nickel sites (Ni°), indicating that carabonate species are further decomposed yielding CO and most possibly also CO_2_ in the gas phase. The weak bands in the *ν* (C-H) region are present on the spectra obtained up to 500 °C (Figure 7A, trace i) implying that CH*_x_* species are thermally stable and remained adsorbed on the catalyst surface.

A similar experiment was conducted over the most active Ni/ZrO_2_ catalyst and the results obtained are presented in Figure 7B. It is observed that the interaction of catalyst with the reformate mixture at 100 °C (trace a) results in the appearance of bands corresponding to bicarbonate species (1640 cm^−1^) [47,48,49], CH_x_ species (2983 and 2966 cm^−1^) [31,36,47] as well as by negative bands (3749 and 3675 cm^−1^) related to the consumption of surface OH groups [31,36,48]. Increase of temperature at 200 °C (trace c) leads to the progressive development of two bands at 1540 and 1425 cm^−1^ due to bicarbonate species [47,48,49]. The intensity of the latter bands increases with increasing temperature up to 300 °C and diminishes upon further heating at 350–400 °C. This is also the case for the band at 1640 cm^−1^, indicating that bicarbonate species are decomposed above 450 °C. At temperatures higher than 400 °C (traces h-i) two new broad bands seem to be developed at ca 1540 and 1350 cm^−1^. The broadness of these bands implies that they may contain contributions from more than one species with their corresponding bands being overlapped.

This can be clearly seen in the spectrum obtained at 500 °C (trace i) where four bands can be clearly discerned located at 1558, 1520, 1370 and 1331 cm^−1^. Those detected at 1520 and 1331 cm^−1^ have been previously attributed to bidentate carbonates [49,50], whereas those located at 1558 and 1370 cm^−1^ can be assigned to bidentate formate species [51] adsorbed on ZrO_2_ surface. The appearance of the latter bands is accompanied by evolution of three peaks in the *ν*(CO) region due to CO linearly adsorbed on reduced Ni sites (2021 cm^−1^) and bridged bonded CO (1909 and 1858 cm^−1^) [43,46,52,53,54].

Interestingly, CH*_x_* species are eliminated from the spectra obtained above 400 °C followed by evolution of CH_4_ in the gas phase, as evidenced by the detection of the 3016 cm^−1^ band (traces h–i). Production of CH_4_ at the expense of CH_x_ species can be clearly seen in Figure 8B where the spectra obtained at 100 and 450 °C in the wavenumber range of 3200–2400 cm^−1^ are presented.

Based on the above it can be suggested that the reaction of steam reforming of propane over Ni/ZrO_2_ catalyst proceeds via a dissociative adsorption of propane on metallic Ni leading to the formation of C_3_H_x_ species, which are further decomposed toward CH_x_ species and probably carbon oxides due to the presence of H_2_O adsorbed on the support surface. This may result in the formation of the bicarbonate species (1640, 1540 and 1425 cm^−1^) detected at low temperatures on the surface of the support [20]. Part of CH_x_ species are hydrogenated above 400 °C, yielding methane in the gas phase (band at 3016 cm^−1^) whereas the rest interact with adsorbed water producing formates (bands at 1558 and 1370 cm^−1^) and, eventually, CO species adsorbed on the Ni surface (bands at 2021, 1909 and 1858 cm^−1^). Formates may also interact with hydroxyl groups producing H_2_ and carbonate species (1520 and 1331 cm^−1^), which are further decomposed to CO_2_ [20]_._ It should be noted, however, that, under certain conditions, CH*_x_* species may be also dehydrogenated producing C and H_2_. Surface carbon is either accumulated on the catalyst surface resulting in catalyst deactivation (which is not the case here) or it interacts with the hydroxyl groups or the lattice oxygen of the support yielding CO or CO_2_ [3,16,19,31,55].

The ability of CH*_x_* species to be converted to methane and/or formates on the surface of Ni/ZrO_2_ may imply that CH*_x_* species are more weakly adsorbed and/or more reactive on the surface of this catalyst, thus resulting in higher overall activity for the propane steam reforming reaction. This agrees well with results of Figure 5, where it was shown that catalytic activity increases progressively with increasing methane selectivity. If as discussed above the origin of adsorbed CO is formate species, the high reactivity of CH*_x_* species over Ni/ZrO_2_ is also indirectly confirmed by the significantly higher population of carbonyl species observed over this catalyst. On the other hand, although CH_x_ species were also detected on the surface of Ni/TiO_2_ catalyst, no band due to gas phase methane or formate species was observed, indicating that CH_x_ species cannot be further converted in the temperature range investigated. The increase of S_CH4_ in parallel with the increase of catalytic activity (Figure 5) indicates that the reactivity of CH_x_ species is strongly related to the conversion of propane to the desired products. Although detailed mechanistic studies should be conducted to further explore the reaction pathway, it can be suggested that CH_x_ species are key reaction intermediates for the reaction of propane steam reforming.

## 4. Conclusions

The results of the present study show that the catalytic activity of Ni and the product distribution for the propane steam reforming reaction depend strongly on the nature of the support. The specific reaction rate measured for Ni/ZrO_2_ catalyst was found to be more than one order of magnitude higher compared to that measured for Ni/TiO_2_. The intermediate production of CH_4_ is strongly influenced by the type of metal oxide used as support following the same trend with that of catalytic activity. DRIFTS studies provided evidences that the most active Ni/ZrO_2_ catalyst is able to convert the intermediate produced CH*_x_* species to CH_4_ and/or formate species and, subsequently, to carbon oxides and H_2_. In contrast, CH*_x_* species seem to be less reactive when Ni is dispersed on TiO_2_, thus resulting in a lower reaction rate. In addition to the high activity of Ni/ZrO_2_ catalyst, it was also found to be stable for more than 30 h on stream and therefore, is a promising candidate for the production of H_2_ for fuel cell applications.

## Figures and Tables

**Table 1:** Physicochemical properties of supported Ni (5 wt.%) catalysts and their apparent activation energies for propane steam reforming reaction.

Catalyst	SSA ^(a)^ (m^2^/g)	d_MxOy_^(b)^ (nm)	D_Ni_^(c)^ (%)	d_Ni_^(c)^ (nm)	Activation Energy (kJ/mol)
5%Ni/ZrO_2_	39	15.0	5.7	17.8	154
5%Ni/YSZ	11	20.9	4.7	21.4	140
5%Ni/Al_2_O_3_	66	6.0	4.0	25.5	121
5%Ni/CeO_2_	39	10.5	11.9	8.5	102
5%Ni/TiO_2_	41	21.8	2.8	36.1	127

^(a)^ Specific surface area, estimated with the BET method. ^(b)^ Primary crystallite size of M*_x_*O*_y_*, estimated from XRD line broadening. ^(c)^ Dispersion and mean particle size of Ni, estimated from selective chemisorption measurements.

**Table 2:** Selected area electron diffraction (SAED) analysis of TEM images obtained for 5%Ni/YSZ, 5%Ni/CeO_2_ and 5%Ni/TiO_2_ catalysts.

Catalyst	Spot	d-Spacing (Å)	Formula	Crystallographic Plane (h k l)	JCPDS No
5%Ni/YSZ	1	3.0	Y_0.15_Zr_0.85_O_1.93_	(111)	30-1468
	2	2.6	Y_0.15_Zr_0.85_O_1.93_	(200)	30-1468
	3	2.1	Ni	(111)	1-1258
5%Ni/CeO_2_	1	3.1	CeO_2_	(111)	1-800
	2	2.7	CeO_2_	(200)	1-800
	3	2.1	Ni	(111)	1-1258
5%Ni/TiO_2_	1	3.5	TiO_2_	(101)	1-562
	2	2.4	TiO_2_	(103)	1-562
	3	2.1	Ni	(111)	1-1258

## Data Availability

Not applicable.
